# A multi‐level exploration of the relationship between temperature and species diversity: Two cases of marine phytoplankton

**DOI:** 10.1002/ece3.9584

**Published:** 2022-12-12

**Authors:** Junfeng Gao, Qiang Su

**Affiliations:** ^1^ College of Earth and Planetary Sciences (CEPS) University of Chinese Academy of Sciences (UCAS) Beijing China

**Keywords:** coccolithophore, dinoflagellate cyst, fractal model, species abundance distribution, the asymptotic relationship

## Abstract

The relationship between temperature (*T*) and diversity is one of the most important issues in ecology. It provides a key direction not only for exploring the determinants of diversity's patterns, but also for understanding diversity's responses to climate change. Previous studies suggested that *T*–diversity relationships could be positive, negative, or unimodal. Although these studies accumulated many informative achievements, they might be unsatisfied due to (1) investigating inadequate range of *T*, (2) selecting incomplete diversity metrics, and (3) making insufficiently detailed analysis of correlation. In this study, species diversity is estimated by four most commonly used diversity metrics and three parameters of species abundance distribution (SAD), and two global datasets of marine phytoplankton (covering a wider range of *T*) are used to evaluate the *T*–diversity relationships according to a piecewise model. Results show that all aspects of diversity (except evenness) have the similar relationship with *T* in the range of lower *T*, noting that diversity significantly increases as *T* increases. However, in the range of higher *T*, diversity may significantly decrease or nearly constant, which indicates that their relationships may be the unimodal or asymptotic. The asymptotic relationship found by this study is assumed that increasing diversity with *T* will gradually approach the Zipf's law (1:1/2:1/3…). If such assumption can be verified by future investigations, the intrinsic mechanism of the asymptotic relationship is likely to be crucial in understanding the *T*–diversity relationships.

## Graphical abstract

Previous studies of *T*–diversity relationships suggested that the *T*–diversity relationships were not always positive or negative and they could be unimodal or otherwise. Thus, this study uses the piecewise model, rather than the method of the overall correlation between *T* and diversity, to explore the details of how exactly diversity changes with *T*. This study assumes the asymptotic relationship is that increasing diversity with *T* will gradually approach the Zipf's law (1:1/2:1/3…).

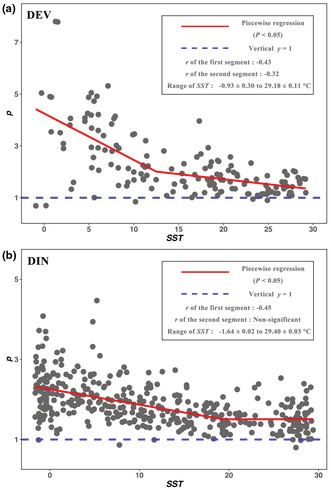

## 1 INTRODUCTION

The relationship between temperature (*T*) and diversity is one of the most fundamental topics in ecology (Brown, 2014; Danovaro et al., 2004; Hawkins et al., 2003; Qian, 2010). Many ecologists and climatologists have long been fascinated by their relationship, as it provides an important path not only for exploring the global pattern of diversity (Currie et al., 2004), such as the latitudinal diversity gradients (Allen et al., 2002; Willig & Presley, 2013), but also for understanding how diversity responds to global climate change (Chaudhary et al., 2021; Yasuhara & Danovaro, 2016). Although numerous theoretical and empirical studies of *T*–diversity relationship have been accumulated over the past century (Hawkins et al., 2003; Peters et al., 2016; Tittensor et al., 2010), there is still largely controversial about the exact pattern of their relationships (Danovaro et al., 2004; O'Hara & Tittensor, 2010; Yasuhara & Danovaro, 2016).

Traditional theories generally suggested that the *T*–diversity relationship was positive (Brown, 2014; Willig & Presley, 2013). On one hand, warmer places were more amenable and hence could support more species (Currie et al., 2004; Tittensor et al., 2010). On the other hand, increasing *T* led to greater diversity by promoting the rates of speciation (Allen et al., 2002, Willig & Presley, 2013). However, the empirical *T*–diversity relationship was not always positive. Many studies showed that their relationships could be unimodal (Chaudhary et al., 2017; Lin et al., 2020; Yasuhara & Danovaro, 2016), which might be explained by a modified version of the physiological tolerance hypothesis (please see Section 4 and Yasuhara & Danovaro, 2016). Additionally, negative relationships were also found, although a general explanation accepted by most ecologist was missing (Danovaro et al., 2004; Grady et al., 2019; O'Hara & Tittensor, 2010). Thus, there is an apparent discrepancy between theoretical studies and empirical investigations. This imposed a challenge for the in‐depth understanding of the *T*–diversity relationship (Brown, 2014; Chaudhary et al., 2021; Yasuhara & Danovaro, 2016).

Such discrepancy may be attributed to the *T* range of datasets, the selections of diversity metric and the analytical methods of correlation (Peters et al., 2016; Stevens & Willig, 2002; Tittensor et al., 2010). Firstly, the range of *T* in some studies might be insufficient to present a complete *T*–diversity relationship (Danovaro et al., 2004; O'Hara & Tittensor, 2010); secondly, using only one diversity metric (generally species richness) to analyze the *T*–diversity relationship (Boltovskoy & Correa, 2017; Hawkins et al., 2003; Qian, 2010) could not reflect other aspects of diversity, such as evenness and dominance (Tittensor et al., 2010; Yasuhara & Danovaro, 2016); Finally, the method of the overall correlation between *T* and diversity (e.g., general linear regression, quadratic regression, and generalized additive model) might lose the details of *T*–diversity relationships, as recent studies suggested that they were inappropriate test for unimodal relationship (Simonsohn, 2018; Tessier, 2020). Thus, there is still a need for the analyses that take into account the wide *T* range of datasets, multiple aspects of diversity and precise methods of correlation (Chaudhary et al., 2021; Willig & Presley, 2013; Yasuhara et al., 2020).

The purpose of this study is to explore a multi‐level understanding of how exactly diversity changes with *T*. To this end, 4571 quantitative community samples from two global phytoplankton datasets (Coccolithophore and Dinoflagellate, please see de Vries et al., 2020; Zonneveld et al., 2013) are used to evaluate their relationship according to a piecewise model (Muggeo, 2003), which can quantitatively test multiple shapes of *T*–diversity relationship (Simonsohn, 2018, Tessier, 2020). The species diversity is estimated by four most commonly used diversity metrics (Pielou, 1975) and three parameters of species abundance distribution (SAD) (McGill et al., 2007), including a new fractal SAD model (Su, 2016).

## 2 METHOD

To understand the *T*–diversity relationship from multiple aspects, four diversity metrics are used, including Species richness (*S*), Shannon's index (*H*′), Simpson's index (*D*), and Pielou's evenness (*J*) (Pielou, 1975). Three parameters of SAD models are also calculated as the diversity surrogates (McGill et al., 2007), including geometric series model (*K*), Zipf model (*γ*), and a new fractal model (*p*) (Su, 2016).

### 2.1 Diversity metrics

*H*′, *D* and *J* are usually expressed as (Pielou, 1975)

(1) $$H^{\prime }=-\sum_{i=1}^{S}p_{i}\ln p_{i}^{2}$$

(2) $$D=1-\sum_{i=1}^{S}p_{i}^{2}$$

(3) $$J=\frac{H^{\prime }}{\ln S}$$

where *p*_*i*_ is the relative abundance of the *i*‐th species (*i* = 1, 2, 3, … *S*). The higher diversity, the higher *H*′ and *J*, and the lower *D* (Pielou, 1975).

### 2.2 The SAD parameters (*K*, *γ* and *p*)

*K* is the parameter of geometric series model, which is derived from an ecological process where each species takes a constant fraction (*K*) of the remaining resources (McGill et al., 2007). The expected abundance *N*_*r*_ of species at rank *r* (= 1, 2, 3, … *S*) can be expressed as

(4) $$N_{r}=N_{T}\;K\;{\left(1-K\right)}^{r-1}$$

where *N*_*T*_ is the total number of individuals in the community; the preemption coefficient *K* is the only estimated parameter which gives the decay rate of abundance per rank (Oksanen et al., 2020). A smaller *K* indicates a greater *S* and a higher evenness (Wang et al., 2021).

Zipf model is generated from the fractal tree (Frontier, 1987) or the spatial model (Harte et al., 1999; Pueyo, 2006). A meta‐analysis of SADs suggested that it was one of the best fitting models (Ulrich et al., 2010). In Zipf model (Oksanen et al., 2020), the expected abundance *N*_*r*_ is

(5) $$N_{r}=N_{T}\;p_{1}r^{-\gamma }$$

where *N*_*T*_ is the total number of individuals in the community; *p*_1_ is the fitted proportion of the most abundant species; *γ* is a decay coefficient that indicated the influence of priority effect (Chen, 2014).

*p* is derived from a fractal model (Su, 2016). According to the fractal hypothesis (when *X* more species appear at each step of the accumulation process, their abundance are *x* times less abundant and *X* = *x* ^*d*^, where *d* is the fractal dimension), SAD in a community can be described as

(6) $$\frac{N_{r}}{N_{1}}=r^{-p}$$

where *r* (= 1, 2, 3, … *S*) is the rank of species sorted down by species abundance; *N*_*r*_ and *N*_1_ are the number of individuals of the *r*‐th and the first species in descending order; *p* (= 1/*d*) is the fractal parameter that determines SAD. For example, when *p* = 1 and *S* = 3, *N*_*r*_/*N*_1_ is 1:1/2:1/3; when *p* = 2 and *S* = 3, *N*_*r*_/*N*_1_ is 1:1/4:1/9. The lower *p*, the higher species diversity (Su, 2016).

Let *F*_*r*_ = ln (*N*_*r*_/*N*_1_) and *D*_*r*_ = ln (*r*). By minimizing the sum of squared error ($\sum_{r=1}^{S}{\left(-pD_{r}-F_{r}\right)}^{2}$), *p* can be expressed as (Su, 2016)

(7) $$p=\frac{-\sum_{r=1}^{S}D_{r}F_{r}}{\sum_{r=1}^{S}D_{r}^{2}}$$

The sum formula of Equation 6 is

(8) $$\frac{N_{T}}{N_{1}}=\sum_{r=1}^{S}r^{-p}$$

where *N*_*T*_ is the total number of individuals in the community.

### 2.3 Datasets

#### 2.3.1 Species diversity

Global Sem Coccolithophore Abundance Compilation Dataset (DEV) (de Vries et al., 2020) and Geographic Distribution of Dinoflagellate Cysts in Surface Sediments Dataset (DIN) (Zonneveld et al., 2013) are used to establish the *T*–diversity relationships. DEV contained 266 species and 2556 community samples, which were compiled from all available scanning electron microscopy marine coccolithophore species abundance observations (de Vries et al., 2020). DIN were based on data from global dinoflagellate cysts in surface sediment samples that have been prepared with a comparable methodology and taxonomy (Zonneveld et al., 2013), including 71 species and 2405 community samples. They are selected here because (1) they are reliable as they have been used in diversity studies (Boyd et al., 2018; de Vries et al., 2021); (2) the published datasets are easy to recheck; (3) they are the global datasets, so their relationships can be evaluated over a wider *T* range; (4) they contain the abundance of each species, by which multiple aspects of species diversity can be calculated; (5) they represent two taxonomic groups (de Vries et al., 2020; Zonneveld et al., 2013), which can identify the similarity of relationships between *T* and diversity.

#### 2.3.2 Temperature

The sea surface temperature (*SST*) data are used to analyze the *T*–diversity relationship. These data are monthly long‐term mean (1981–2010) from NOAA optimum interpolation *SST* analysis (https://psl.noaa.gov/data/gridded/data.noaa.oisst.v2.html#source).

#### 2.3.3 The relationship between temperature and species diversity

*S*, *H′*, *D*, *J*, *γ*, and *K* are calculated by the vegan and sads packages (Oksanen et al., 2020; Prado et al., 2014). The fractal *p* are calculated by Equation 7. All diversity metrics and *SST* are standardized by taking the average for 5° × 5° grids. The relationships between average *SST* and diversity metrics are divided into two segments according to the piecewise regressions models using the segmented package (Muggeo, 2003). The temperatures at breakpoints between segments (*T*_bp_) are marked by the piecewise models. The directions of segments are measured by Pearson's correlation coefficient (*r*). According to the definition of unimodality (Tessier, 2020; Yasuhara & Danovaro, 2016), the *T*–diversity relationship is unimodal if the directions of *r* in two segments are significantly opposite. The rate of change is calculated by the slope of regression (*m*). Significance of the *T*–diversity relationship in each segment is assessed by *p* (at .05 level).

In Supplemental Files (Table S1; Figures S1–S4), the relationships between *SST* and diversity metrics are fitted by the method of the overall correlation, including general linear regression, quadratic regression, and generalized additive model (GAM). The differences of fitting between the piecewise regressions and other models are compared by the coefficient determination (*R* ^2^) and Akaike information criterion (AIC). *R* ^2^ and AIC denotes how well they fit the empirical *T*–diversity relationships. The larger *R* ^2^ and the smaller AIC, the better performance (Muggeo, 2003). Additionally, since the sample distributions of two datasets are uneven at the different latitudinal ranges (56% of the samples of DEV and 41% of the samples of DIN are from 30 to 50°N/S), bootstrap simulations (Boltovskoy & Correa, 2017; Saupe et al., 2019) are used by (1) randomly selecting 5 original samples from each 5° latitude bands, (2) taking the average of *SST* and diversity metrics for 5° × 5° grids, and (3) calculating piecewise regression of *T*–diversity relationship. These three steps are repeated 500 times (with replacement) to yield the average *r*, by which the *T*–diversity relationships are evaluated. The results of bootstrap simulations are listed in Table S2.

All statistical analyses in this paper are performed in R ver. 4.0.4 (www.r‐project.org), and code can be archived in figshare (Gao & Su, 2022).

## 3 RESULTS

**FIGURE 1:**
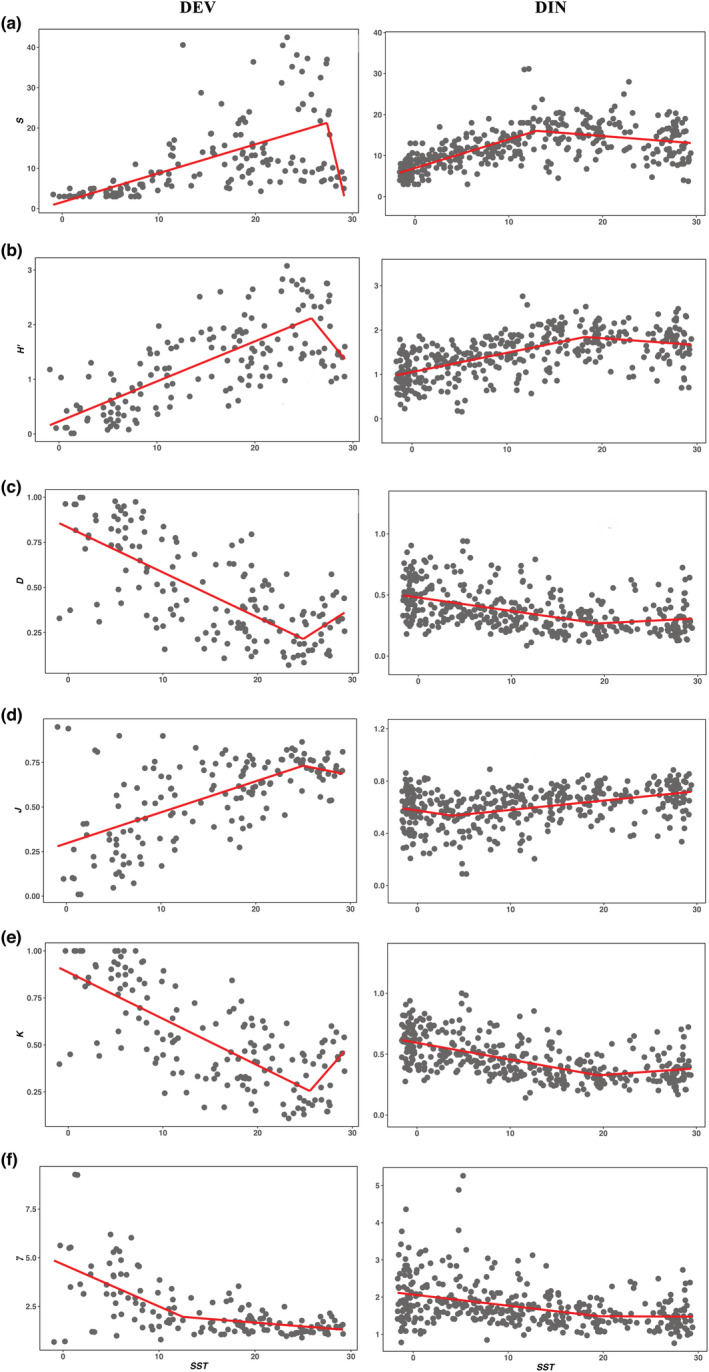
The *T*–diversity relationships for DEV and DIN. They are measured by (a) species richness (*S*), (b) Shannon's index (*H′*), (c) Simpson's index (*D*), (d) Pielou's evenness (*J*), (e) geometric series model' parameter (*K*), and (f) Zipf model's parameter (*γ*). The red line represents the regression result of the piecewise model. The arrows mark the non‐significant *T*–diversity relationship in the piecewise model.

The sea surface temperature (*SST*) matched with Global Sem Coccolithophore Abundance Compilation Dataset (DEV) is from 0.93 ± 0.30 to 29.18 ± 0.11°C, and *SST* of Geographic Distribution of Dinoflagellate Cysts in Surface Sediments Dataset (DIN) is from −1.63 ± 0.02 to 29.40 ± 0.03°C. For DEV, *T*_bp_ of four metrics (*S*, *H′*, *D* and *J*) are 27.39, 25.77, 24.82, and 24.99°C (Table 1). For DIN, *T*_bp_ of four metrics are 12.93, 18.20, 19.28, and 3.7°C (Table 1). In the first segment of the piecewise regression (Figure 1), *S* and *H′* of two datasets are significantly positive with *SST* (DEV: *r*_*S*1_ = 0.60, *m*_*S*1_ = 0.72, *r*_*H′*1_ = 0.73, *m*_*H′*1_ = 0.07; DIN: *r*_*S*1_ = 0.73, *m*_*S*1_ = 0.70, *r*_*H′*1_ = 0.56, *m*_*H′*1_ = 0.04). *D* of two datasets are significantly negative with *SST* (DEV: *r*_*D*1_ = −0.69, *m*_*D*1_ = −0.02; DIN: *r*_*D*1_ = −0.41, *m*_*D*1_ = −0.01). For DEV, *J* is significantly correlated with *SST* (*r*_*J*1_ = 0.55, *m*_*J*1_ = 0.02), but the *T*–*J* relationship is not significant for DIN. In the second segment (Figure 1), *S* of two datasets show the significant correlation with *SST* (DEV: *r*_*S*2_ = −0.75, *m*_*S*2_ = −10.20; DIN: *r*_*S*2_ = −0.22, *m*_*S*2_ = −0.18). *H′* and *D* of two datasets are not significant (Table 1). *J* has a significant correlation with *SST* for DIN (*r*_*J*2_ = 0.41, *m*_*J*2_ = 0.01), but not for DEV.

**TABLE 1:** The temperature at the breakpoint (*T*_bp_) and the correlation between *T* and diversity

Datasets	Diversity metric	*T*_bp_ (°C)	*r*_1_	*m*_1_	*P*_1_	*r*_2_	*m*_2_	*P*_2_
DEV	Species richness (*S*)	27.39	0.60	0.72	<.05	−0.75	−10.20	<.05
	Shannon's index (*H′*)	25.77	0.73	0.07	<.05	−0.39	−0.22	.06
	Simpson's index (*D*)	24.82	−0.69	−0.02	<.05	0.34	0.03	.07
	Pielou's evenness (*J*)	24.99	0.55	0.02	<.05	−0.17	−0.01	.40
	Geometric series model' parameter (*K*)	25.51	−0.72	−0.02	<.05	0.39	0.06	.07
	Zipf model's parameter (*γ*)	12.48	−0.42	−0.22	<.05	−0.33	−0.04	<.05
	Fractal *p*	12.49	−0.43	−0.18	<.05	−0.32	−0.04	<.05
DIN	Species richness (*S*)	12.93	0.73	0.70	<.05	−0.22	−0.18	<.05
	Shannon's index (*H′*)	18.20	0.56	0.04	<.05	−0.15	−0.02	.10
	Simpson's index (*D*)	19.28	−0.41	−0.01	<.05	0.10	<0.01	.30
	Pielou's evenness (*J*)	3.7	−0.90	−0.01	.35	0.41	0.01	<.05
	Geometric series model' parameter (*K*)	19.44	−0.54	−0.01	<.05	0.15	0.01	.15
	Zipf model's parameter (*γ*)	19.54	−0.31	−0.03	<.05	<0.01	<0.01	.93
	Fractal *p*	19.44	−0.45	−0.04	<.05	<0.01	<0.01	.94

*Note*: *T* at the breakpoint marks a change in direction from one segment to another. *m*_1_ and *m*_2_ are the slope on the first and second segments of the piecewise model. *r*_1_ and *r*_2_ represent the correlation coefficients on the two segments of the piecewise model. *P*_1_ and *P*_2_ represent their significance, respectively. When *p* < .05, their correlation is significant.

**FIGURE 2:**
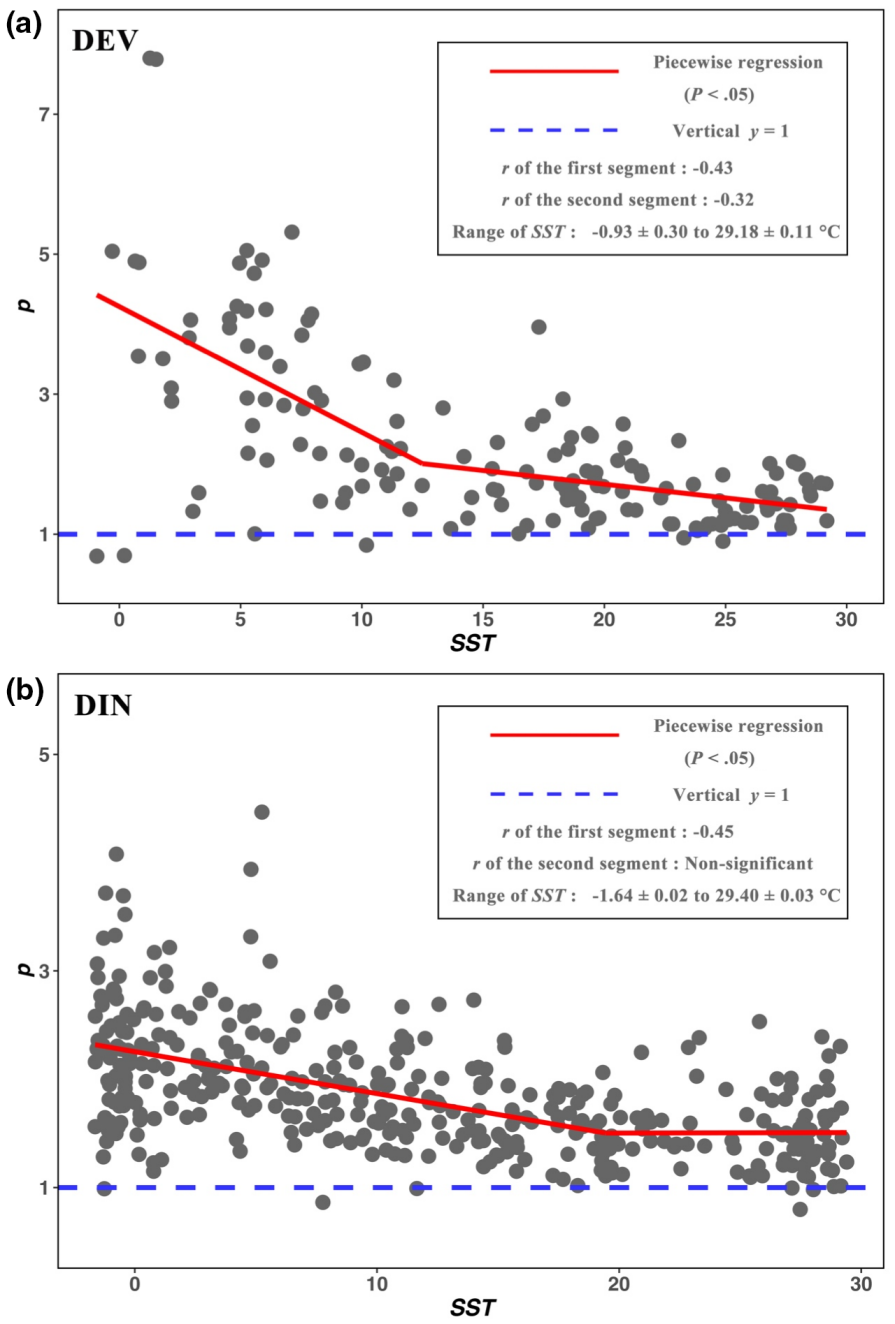
The relationships between *T* and fractal *p* for DEV (a) and DIN (b). The red line represents the regression result of the piecewise model. The box in the figure gives the details of *T*–*p* relationship in the piecewise model.

*T*_bp_ of *K* are 25.51 and 19.44°C for DEV and DIN, respectively. When *SST* < *T*_bp_, *K* of two datasets significantly decrease with *SST* (DEV: *r*_*K*1_ = −0.72, *m*_*K*1_ = −0.02; DIN: *r*_*K*1_ = −0.54, *m*_*K*1_ = −0.01). When *SST* > *T*_bp_, their correlation are positive (DEV: *r*_*K*2_ = 0.39, *m*_*K*2_ = 0.06; DIN: *r*_*K*2_ = 0.15, *m*_*K*2_ = 0.01), but not significant (Table 1). *T*_bp_ of *γ* and *p* are 12.48 and 12.49°C for DEV, and they are 19.54 and 19.44°C for DIN. In the first segment, *γ* and *p* of two datasets significantly decrease with *SST* (DEV: *r*_*γ*1_ = −0.42, *m*_*γ*1_ = −0.22, *r*_*p*1_ = −0.43, *m*_*p*1_ = −0.18; DIN: *r*_*γ*1_ = −0.31, *m*_*γ*1_ = −0.03, *r*_*p*1_ = −0.45, *m*_*p*1_ = −0.04). In the second segment, *γ* and *p* of DEV significantly decrease with *SST* (*r*_*γ*2_ = −0.33, *m*_*γ*2_ = −0.04, *r*_*p*2_ = −0.32, *m*_*p*2_ = −0.04), but *γ* and *p* of DIN do not significantly change with *SST*. *γ* and *p* of two datasets all tend to be flat (Figure 2).

## 4 DISCUSSION

Since the mid‐20th century, many ecologists have sought to understand the relationship between *T* and diversity (Allen et al., 2002; Brown, 2014; Hawkins et al., 2003; Qian, 2010). As empirical investigations were accumulated (Costello et al., 2015; Tittensor et al., 2010), it was known that *T*–diversity relationships were not always positive (Chaudhary et al., 2021; Lin et al., 2020) and they could be negative or unimodal (Chaudhary et al., 2021; Yasuhara & Danovaro, 2016). The biggest difference of this study is that the piecewise model, rather than the method of the overall correlation, is used to explore the details of how exactly diversity changes with *T* (Muggeo, 2003). Although the overall correlation has been widely used in previous studies (Peters et al., 2016; Stevens & Willig, 2002; Tittensor et al., 2010), the piecewise model may be more suitable. Firstly, some methods of overall correlation (e.g., general linear regression) can be considered a special case of the piecewise model (Muggeo, 2003). Secondly, since the *T*–diversity relationship could be unimodal (Chaudhary et al., 2021; Lin et al., 2020; Yasuhara et al., 2020; Yasuhara & Danovaro, 2016), the overall correlation method (e.g., general linear regression, quadratic regression and GAM) could not adequately identify such pattern (Simonsohn, 2018; Tessier, 2020). Thirdly, the unimodality and more details of the *T*–diversity relationships (e.g., the temperatures at breakpoint [*T*_bp_]) can be shown according to the piecewise model (Table 1) (Simonsohn, 2018, Tessier, 2020). Finally, in this study, piecewise model provides a better or similar quality of fit than other regressions (please see Table S1 and Figures S1–S4).

As mentioned above, traditional ecological theories usually suggested a positive *T*–diversity relationship, which was apparently inconsistent with the negative and unimodal relationships observed by many investigations (Chaudhary et al., 2021; Lin et al., 2020; Yasuhara & Danovaro, 2016). In fact, the discrepancy between theoretical and empirical studies is not only reflected in the results of *T*–diversity relationships (Currie et al., 2004; Danovaro et al., 2004; Yasuhara & Danovaro, 2016), but also in the understanding of their relationships (Allen et al., 2002; Brown, 2014; Hawkins et al., 2003). Numerous empirical studies indicated that different taxa might have quite dissimilar *T*–diversity behaviors (Boltovskoy & Correa, 2017; Grady et al., 2019; Jablonski et al., 2017; Snelgrove et al., 2017). For example, Boltovskoy and Correa (Boltovskoy & Correa, 2017) revealed that the *T*–diversity relationship was unimodal for foraminifera, and it was completely positive for radiolaria. Grady et al. (2019) reported that diversity of sharks and fish constantly increased with *T*, but mammal and bird generally showed the unimodal *T*–diversity relationships. Snelgrove et al. (2017) found that the diversity of seals was negative with *T*, while the *T*–diversity relationships of most marine taxa were positive or unimodal. Such dissimilar *T*–diversity relationships can also be found in this study (Table 1). Firstly, the *T*–*J* relationships in the first segment are positive for Global Sem Coccolithophore Abundance Compilation Dataset (DEV) and negative for Geographic Distribution of Dinoflagellate Cysts in Surface Sediments Dataset (DIN) (Table 1), noting that the directions of their relationships are entirely opposite. Secondly, the *T*_bp_ of dominance for dinoflagellate is nearly 5°C higher than that for coccolithophore (Table 1). Finally, the coccolithophore richness changes more rapidly with *T*, because *m*_*S*2_ of DEV is an order of magnitude higher than that of DIN (please see the second segments of Table 1). Thus, the differences of *T*–diversity relationships among taxa are undeniable.

However, on one hand, some patterns of *T*–diversity relationships (e.g., positive or unimodal) have been repeatedly found in a wide spectrum of living and fossil marine and terrestrial taxa (Brayard et al., 2005; Yasuhara & Danovaro, 2016). On the other hand, some basic principles (e.g., the metabolic theory of ecology) can actually be applied to different taxonomic groups (Allen et al., 2002; Brown et al., 2004; Hawkins et al., 2007). Accordingly, theoretical ecologists reasonably expect a general *T*–diversity relationship derived by some principles (Allen et al., 2002; Brown, 2014; Willig & Presley, 2013). The object of this study is not to identity which *T*–diversity relationship is the general one, but simply to provide valuable options for the future exploration. Actually, since some metrics clearly present the consistent patterns for two datasets (Table 1; Figures 1 and 2), the following discussion will focus on the similarity of *T*–diversity relationships.

Firstly, in the first segment of the piecewise model, *S* and *H′* of two datasets significantly increase with *T*, and their *D*, *K*, *γ*, and *p* significantly decrease with *T* (Table 1). As *S* and *H′* are positive with diversity, and *D*, *K*, *γ*, and *p* are negative with diversity (Pielou, 1975; Su, 2016; Wang et al., 2021), all metrics (except evenness, Figure 1d) are positive with *T*. Such results were also found by many empirical studies (Allen et al., 2002; Peters et al., 2016; Tittensor et al., 2010), and they were consistent with the expectation of traditional theories (Willig & Presley, 2013). For example, the physiological tolerance hypothesis proposed that diversity was higher in warmer places as it provided more tolerate conditions than colder places (Currie et al., 2004). The metabolic hypothesis stated that higher *T* enhanced metabolic efficiency and thus resulted in an increased speciation rate and species diversity (Tittensor et al., 2010). Thus, this study suggests that the *T*–diversity relationships are more likely to be positive at least in the range of lower *T* (the first segment of Figures 1 and 2).

Secondly, since most metrics present positive *T*–diversity relationship at lower *T* (Figure 1; Table 1), the accurate understanding of the *T*–diversity relationship largely depends on how to recognize their relationships at higher *T* (the second segment). In the second segment, some metrics may significantly decrease or nearly constant with *T* (Figures 1 and 2; Table 1), which are substantially different from the traditional theories that are adequately verified in the first segment. Similar results have been frequently observed (Chaudhary et al., 2021; Danovaro et al., 2004; O'Hara & Tittensor, 2010). For example, gastropods, bivalves, and ophiuroids did not show the positive *T*–diversity relationships at higher *T* (O'Hara & Tittensor, 2010; Yasuhara & Danovaro, 2016). To show the details of each metrics changing with *T*, this study classifies the *T*–diversity relationships at higher *T* into three types, noting that significantly negative, non‐significant, and asymptotic.

1. *Significantly negative relationship*: The second segment of the piecewise model shows that *S* significantly decreases with *T*, which are consistent with the bootstrap results (please see Table S2). This means that the direction of variation of *S* is opposite in two segments, which echoes recent studies that considered a wide *T* range (Chaudhary et al., 2021; Lin et al., 2020; Yasuhara et al., 2020; Yasuhara & Danovaro, 2016). Yasuhara and Danovaro (2016) investigated deep‐sea species diversity and indicated that the *T*–*S* relationship was usually unimodal. Chaudhary et al. (2021) found that *S* of most benthic and pelagic taxonomic groups exhibited the significant unimodality. Yasuhara and Danovaro (2016) stated that a modified version of the physiological tolerance hypothesis might explain the unimodal relationships, noting that few species could physiologically tolerate conditions in extremely cold or warm places (Currie et al., 2004; Yasuhara & Danovaro, 2016). This study supports their ideas that the *T*–*S* relationship is more likely to be unimodal when a wider *T* range is considered (Yasuhara & Danovaro, 2016) (Table 1; Figure 1a).
2. *Negative but non‐significant relationship*: In the second, *H′* decreases with *T* (DEV: *r*_*H′*2_ = −0.39; DIN: *r*_*H′*2_ = −0.15), and *D* and *K* increase with *T* (DEV: *r*_*D*2_ = 0.34, *r*_*K*2_ = 0.39; DIN: *r*_*D*2_ = 0.10, *r*_*K*2_ = 0.15). Similar results are also supported by Table S2. However, although three metrics (*H′*, *D* and *K*) present the unimodal relationships, their *T*–diversity relationships are not completely the same with that of *S*. Firstly, their relationships are not significant (DEV and DIN: *p* > .05, Table 1). This was consistent with the studies of terrestrial and aquatic systems (Blanco et al., 2012; Stevens & Willig, 2002). Secondly, the *T*_bp_ of three metrics in DIN all exceed that of *S* by more than 5°C (Table 1). This means that these metrics might not be adequately reflected by the variation of *S*, although they were considered to be partly dependent on *S* (Gosselin, 2006; Stirling & Wilsey, 2001). Finally, current theories rarely predict or explain the patterns of variation of these metrics with *T* (Brown, 2014, Willig & Presley, 2013). Accordingly, it is difficult to draw accurately conclusions about how they change with *T*. This study suggests that the identification of these relationships and their underlying mechanisms are worth further studying.
3. *Asymptotic relationship*: The *T*–diversity relationships described by *γ* and *p* seem to be very different from the relationships presented by other metrics (Figures 1 and 2). *γ* and *p* significantly decrease as *T* increases in the first segment (Table 1), and they slightly decrease (DEV: *m*_*γ*2_ = −0.04, *m*_*p*2_ = −0.04) or remain nearly constant (DIN: *m*_*γ*2_ < 0.01, *m*_*p*2_ < 0.01) in the second segment (Figures 1 and 2). This indicates that *γ* and *p* appear to be flattening with increasing *T* (Figures 1 and 2). When *γ* or *p* approaches 1, SAD (*N*_*r*_/*N*_1_) will be 1:1/2:1/3… (Equation 6 and Frontier, 1987). This was consistent with Zipf's law (Zipf, 1949), which could be the general pattern of SAD (Su, 2018). Therefore, this study supposes that *γ* and *p* with increasing *T* are likely to decrease and approach 1. If the asymptotic pattern can be supported by more studies in future, their intrinsic mechanisms will be crucial in understanding *T*–diversity relationships.

Before the asymptotic pattern is discussed, three points need to be known. Firstly, although Zipf model and fractal model have similar model formulas, their parameters (*γ* and *p*) are derived from different theoretical processes and calculation method (Frontier, 1987; Harte et al., 1999; Pueyo, 2006; Su, 2016). Secondly, the previous studies of the Zipf model have not yet shown any general patterns of diversity (Frontier, 1987; McGill et al., 2007; Mouillot et al., 2000; Ulrich et al., 2010), while the study of fractal model found that *p* approaching 1 might be the general SAD supported by nearly 20,000 community samples (Su, 2018). Thirdly, two hypotheses elucidated the general SAD (Su, 2018) may provide a perspective for the asymptotic relationship between *T* and *p*. H_1_: Species diversity was determined by the entropy that increased with the energy transformation. H_2_: The total assimilated energy of the community (*E*_*T*_) was finite. Thus, this study will try to explain the *T*–*p* relationships according to these hypotheses.

A possible explanation that *p* decreases and asymptotically approaches 1 can be understanded as follows. (1) H_1_ indicates that increasing entropy with *T* will promote higher *N*_*T*_/*N*_1_, as *N*_*T*_/*N*_1_ (Equation 8) is an effective number of species diversity that is related to Rényi's entropy (Hill, 1973; Rényi, 1961). This means that *p* will decrease with increasing *T* (Equation 8). (2) H_2_ indicates that *N*_*T*_/*N*_1_ is finite as *N*_*T*_ is usually equivalent to *E*_*T*_ (Brown, 2014; Hutchinson, 1959). The finiteness of *N*_*T*_/*N*_1_ leads to *p* that ought to be higher than 1 (*N*_*T*_/*N*_1_ converges when *p* > 1, Equation 8). Thus, the combined effect of *H*_1_ and *H*_2_ will contributes to an asymptotic *T*–*p* relationship, and the theoretical minimum *p* is 1. Since a smaller *p* indicates a greater diversity (Su, 2016), *p* = 1 will be the theoretical maximum of diversity presented by the fractal model.

Finally, it should be noted that the results and conclusions of this study are all based on the premise that two datasets are accurate and representative. In fact, although two datasets have been used in many studies of diversity (Boyd et al., 2018; de Vries et al., 2021), they may still have some potential biases. For DEV, some samples from the deeper depth where the light‐dependent coccolithophorids are unlikely to thrive may represent dead and sinking individuals, rather than living ranges (de Vries et al., 2021). For DIN, some morphologically similar species (e.g., *A. tamarense* and *Alexandrium acatenella*; Zonneveld et al., 2013) that are difficult to determine may lead to erroneous records. Additionally, since the responses of terrestrial and aquatic organisms to *T* might greatly differ (such as body size) (Forster et al., 2012), the results found by aquatic groups may be hard to apply to terrestrial communities. Thus, in the future, it is very necessary to test the precise details of the unimodal (*T*–*S*) and asymptotic (*T*–*p*) relationships by more investigations.

## 5 CONCLUSION

1. This study suggests that the relationships between *T* and diversity (including richness, dominance and SAD) are positive at least in the range of lower *T* (Figures 1 and 2; Table 1). This point is consistent with many empirical and theoretical studies (Brown, 2014; Danovaro et al., 2004; O'Hara & Tittensor, 2010; Willig & Presley, 2013).
2. However, in the range of higher *T*, their relationships are significantly negative (*S*) or nearly constant (*γ* and *p*). This indicates that the whole *T*–diversity relationship will be unimodal or asymptotic (Figures 1 and 2; Table 1).
3. As with the frequently discussed unimodal relationship (Chaudhary et al., 2021, Lin et al., 2020, Yasuhara et al., 2020, Yasuhara & Danovaro, 2016), the asymptotic patterns (decreasing *p* with *T* approaches 1) presented by the fractal models (Su, 2016) are worth studying. If the asymptotic relationship is supported by more investigations, this finding is likely to be crucial in understanding the *T*–diversity relationship, global pattern of diversity and the determinisms of species diversity.

## AUTHOR CONTRIBUTIONS

**Junfeng Gao:** Data curation (lead); methodology (lead); resources (equal); software (lead); writing – original draft (equal). **Qiang Su:** Conceptualization (lead); project administration (lead); resources (equal); software (supporting); supervision (lead); writing – original draft (equal); writing – review and editing (lead).

## CONFLICT OF INTEREST

We declare there is no conflict of interest.

## Supporting information


Appendix S1

## Data Availability

Dataset of biological community data for this research (the file “dev.csv” and “din.csv”) can be found in https://doi.pangaea.de/10.1594/PANGAEA.922933 and https://doi.pangaea.de/10.1594/PANGAEA.818280. Sea surface temperature data used during this study (the file “sst.ltm.1981‐2010.nc”, it can be read by Code 1 and Code 2) are openly available from the NOAA/OAR/ESRL PSL, Boulder, Colorado, USA at https://psl.noaa.gov/cgi‐bin/db_search/DBSearch.pl?Dataset=NOAA+Optimum+Interpolation+(OI)+SST+V2&Variable=Sea+Surface+Temperature. The data and codes supporting the results in the paper have archived in figshare https://doi.org/10.6084/m9.figshare.19625328.v6.
