# Supplementary material for: A multi‐level exploration of the relationship between temperature and species diversity: Two cases of marine phytoplankton
Source: Ecol Evol. 2022 Dec 12;12(12):e9584. doi: 10.1002/ece3.9584 (PMC9745263; doi:10.1002/ece3.9584)

STable 1 Comparison of goodness of fit (R^2^) and Akaike information criterion (AIC) between piecewise regression (pie), general linear regression (lr), non-linear quadratic regression (nlr) and generalized additive regression (gam). ΔAIC represent the difference between AIC of piecewise regression and other models (AIC_pie_ - AIC_other models_). When ΔAIC is less than 0, piecewise regression provides a better fit to the empirical data than other models.

STable 2 Average correlation coefficients (*r*) and the proportion of significance (*P* < 0.05) for 500 bootstrap simulations. "*" marks the result inconsistent with the direction calculated using the method of main text.

STable 1

| Taxon | Diversity metric | *R*^2^_pie_ | *R*^2^_lr_ | *R*^2^_nlr2_ | *R*^2^_gam_ | ΔAIC_lr_ | ΔAIC_nlr2_ | ΔAIC_gam_ |
| --- | --- | --- | --- | --- | --- | --- | --- | --- |
| DEV | *S* | 0.37 | 0.28 | 0.30 | 0.39 | -16.61 | -13.99 | -1.93 |
|  | *H'* | 0.52 | 0.48 | 0.50 | 0.57 | -13.52 | -9.76 | 5.45 |
|  | *D* | 0.51 | 0.48 | 0.50 | 0.55 | -6.96 | -2.81 | 7.29 |
|  | *J* | 0.37 | 0.36 | 0.35 | 0.39 | 1.64 | 0.16 | 3.32 |
|  | *K* | 0.53 | 0.49 | 0.49 | 0.56 | -9.32 | -5.18 | 13.33 |
|  | *y* | 0.43 | 0.37 | 0.41 | 0.44 | -9.42 | -0.59 | 0.84 |
|  | *p* | 0.44 | 0.37 | 0.43 | 0.46 | -8.08 | -0.69 | 3.44 |
| DIN | *S* | 0.46 | 0.27 | 0.46 | 0.47 | -112.7 | 4.69 | 2.47 |
|  | *H'* | 0.37 | 0.30 | 0.36 | 0.37 | -34.95 | -4.03 | -0.45 |
|  | *D* | 0.22 | 0.19 | 0.21 | 0.22 | -10.7 | -1.74 | 0.06 |
|  | *J* | 0.14 | 0.12 | 0.12 | 0.15 | -6.81 | -7.62 | 3.55 |
|  | *K* | 0.35 | 0.30 | 0.33 | 0.34 | -22.97 | -4.37 | -0.11 |
|  | *y* | 0.16 | 0.15 | 0.15 | 0.16 | 0.04 | -0.8 | 3.98 |
|  | *p* | 0.29 | 0.27 | 0.28 | 0.29 | -6.04 | -1.46 | 1.24 |

STable 2

| Taxon | Metric | *r*_1_ | *Proportion of significant results* (*P* < 0.05) | *r*_2_ | *Proportion of significant results* (*P* < 0.05) |  |  |  |  |  |
| --- | --- | --- | --- | --- | --- | --- | --- | --- | --- | --- |
| DEV | *S* | 0.51 | 100% | -0.63 | 58.40% |  |  |  |  |  |
|  | *H'* | 0.64 | 99.19% | -0.38 | 12.32% |  |  |  |  |  |
|  | *D* | -0.43 | 80.88% | 0.04 | 21.56% |  |  |  |  |  |
|  | *J* | 0.05 | 38.27% | 0.31* | 62.65% |  |  |  |  |  |
|  | *K* | -0.44 | 83.63% | 0.13 | 22.86% |  |  |  |  |  |
|  | *y* | -0.27 | 40.23% | -0.26 | 52.19% |  |  |  |  |  |
|  | *p* | -0.29 | 49.56% | -0.19 | 49.85% |  |  |  |  |  |
| DIN | *S* | 0.70 | 100% | -0.25 | 3.76% |  |  |  |  |  |
|  | *H'* | 0.52 | 98.40% | -0.05 | 2.80% |  |  |  |  |  |
|  | *D* | -0.40 | 90.36% | 0.03 | 6.00% |  |  |  |  |  |
|  | *J* | 0.06* | 27.73% | 0.29 | 65.10% |  |  |  |  |  |
|  | *K* | -0.49 | 98.00% | 0.03 | 3.4% |  |  |  |  |  |
|  | *y* | -0.23 | 57.11% | -0.08 | 27.64% |  |  |  |  |  |
|  | *p* | -0.32 | 80.68% | -0.05* | 15.09% |  |  |  |  |  |

SFigure 1 The quadratic regression results for the *T* - diversity relationships (blue line with grey 95 % confidence limits), which are estimated by (a) Species Richness (*S*), (b) Shannon’s index (*H'*), (c) Simpson’s index (*D*), (d) Pielou’s evenness (*J*), (e) Geometric series model’ parameter (*K*) and (f) Zipf model’s parameter (*γ*).

SFigure 2 The quadratic regression results for the relationships between *T* and fractal *p* (blue line with grey 95 % confidence limits).

SFigure 3 The *T* - diversity relationships establish by generalized additive model (gam, blue line with grey 95 % confidence limits). They are estimated by (a) Species Richness (*S*), (b) Shannon’s index (*H'*), (c) Simpson’s index (*D*), (d) Pielou’s evenness (*J*), (e) Geometric series model’ parameter (*K*) and (f) Zipf model’s parameter (*γ*).

SFigure 4 The relationships between *T* and fractal *p* establish by generalized additive model (gam, blue line with grey 95 % confidence limits).

SFigure 1


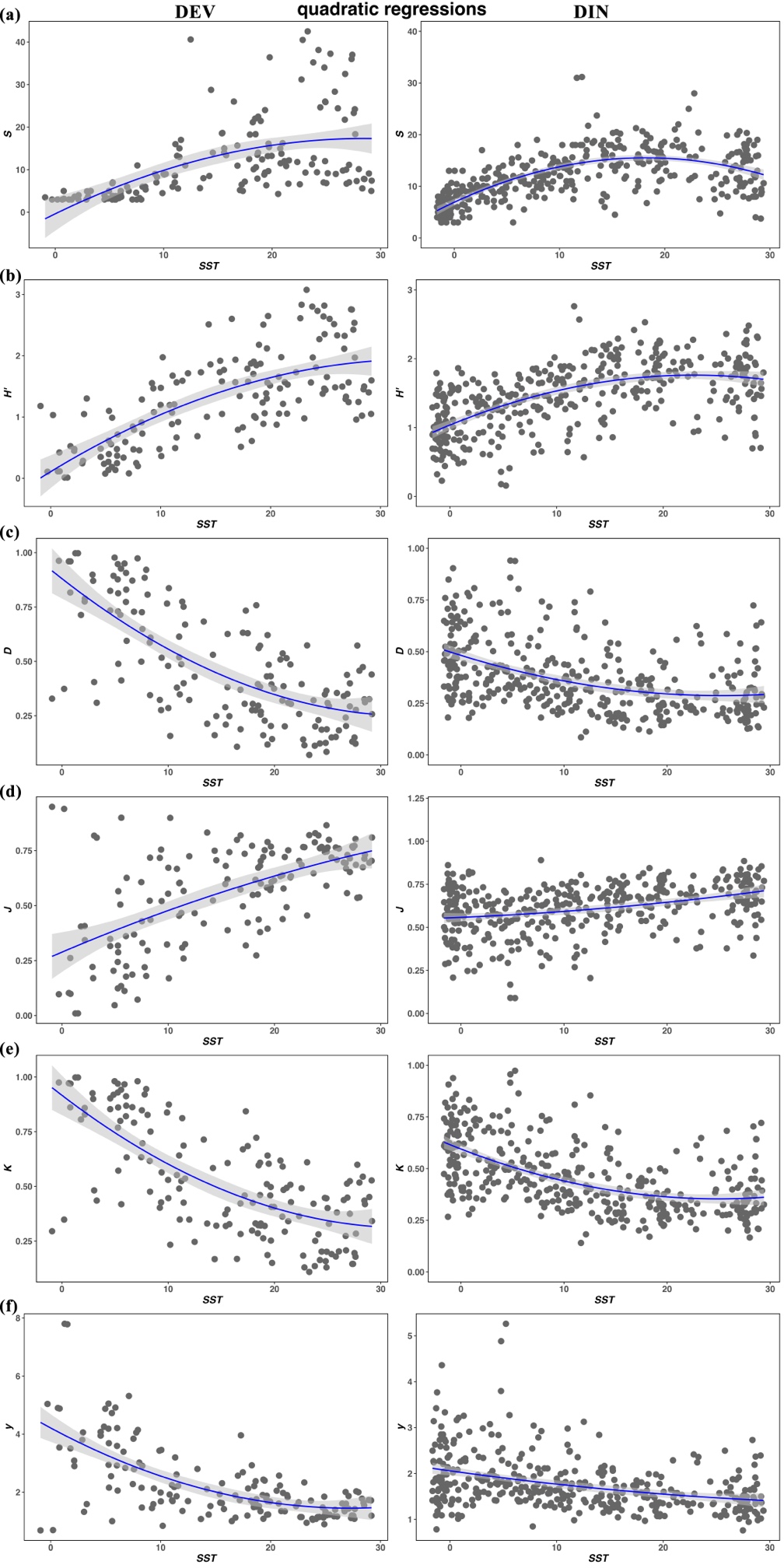


SFigure 2


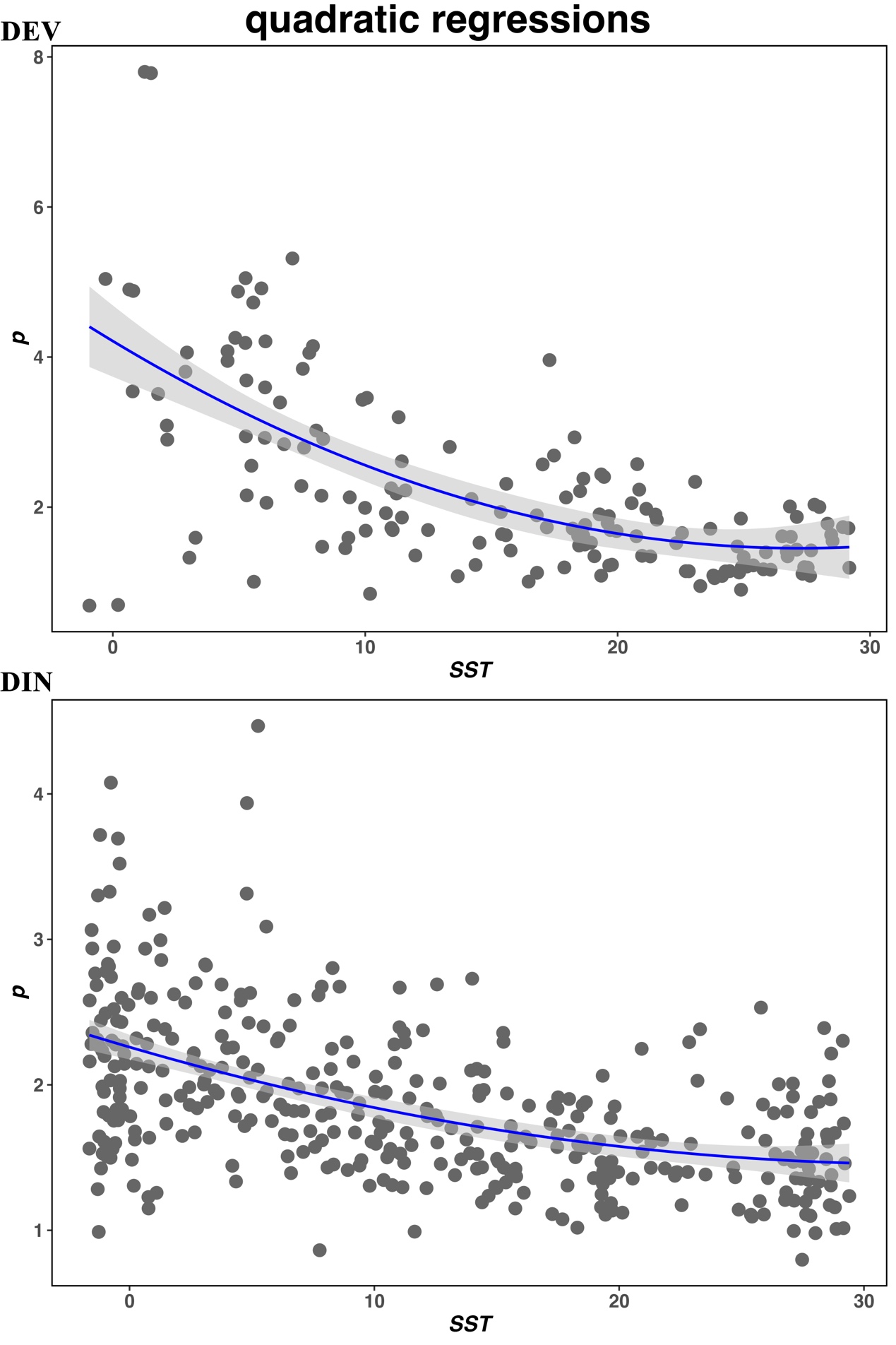


SFigure 3


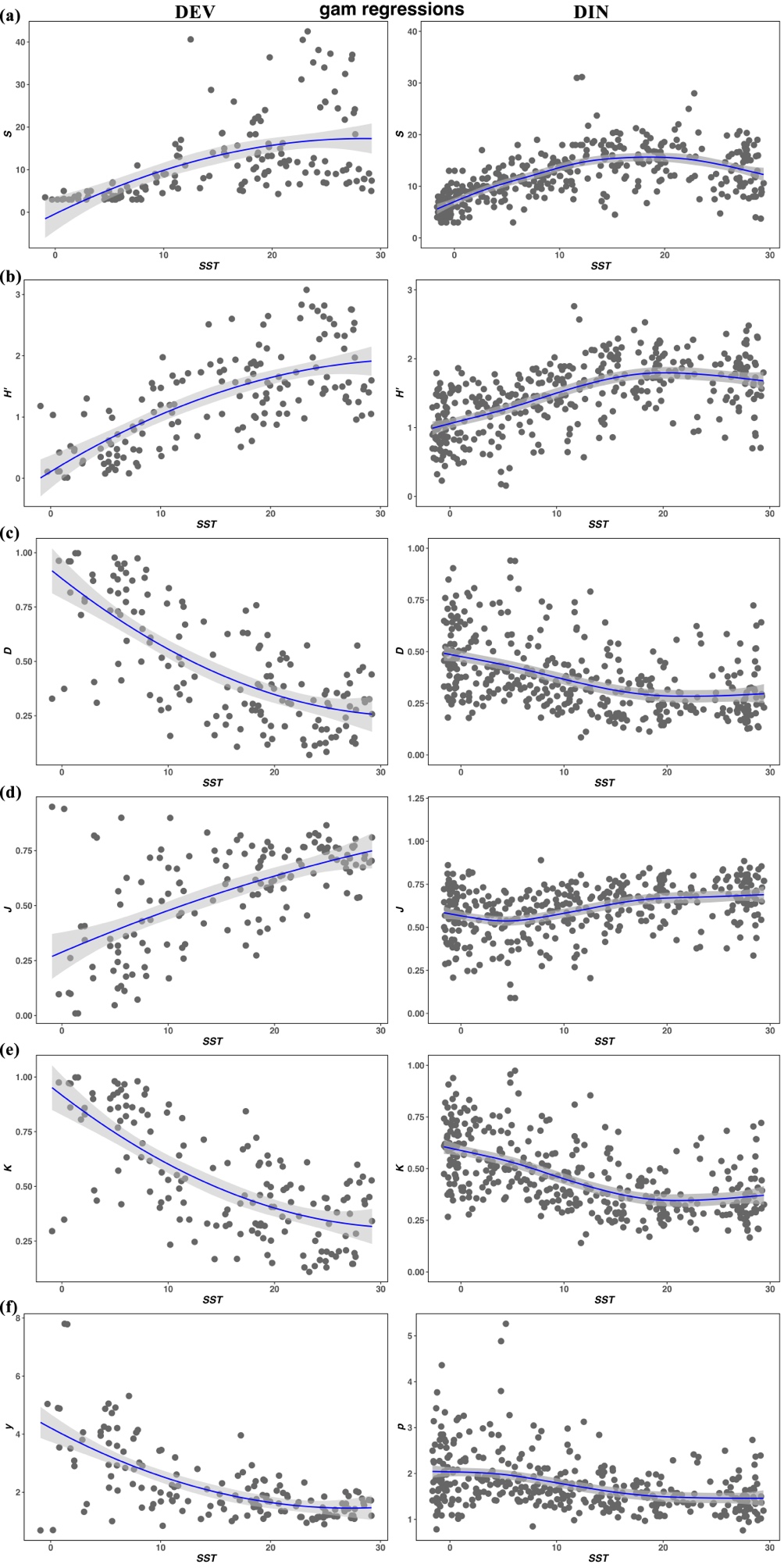


SFigure 4


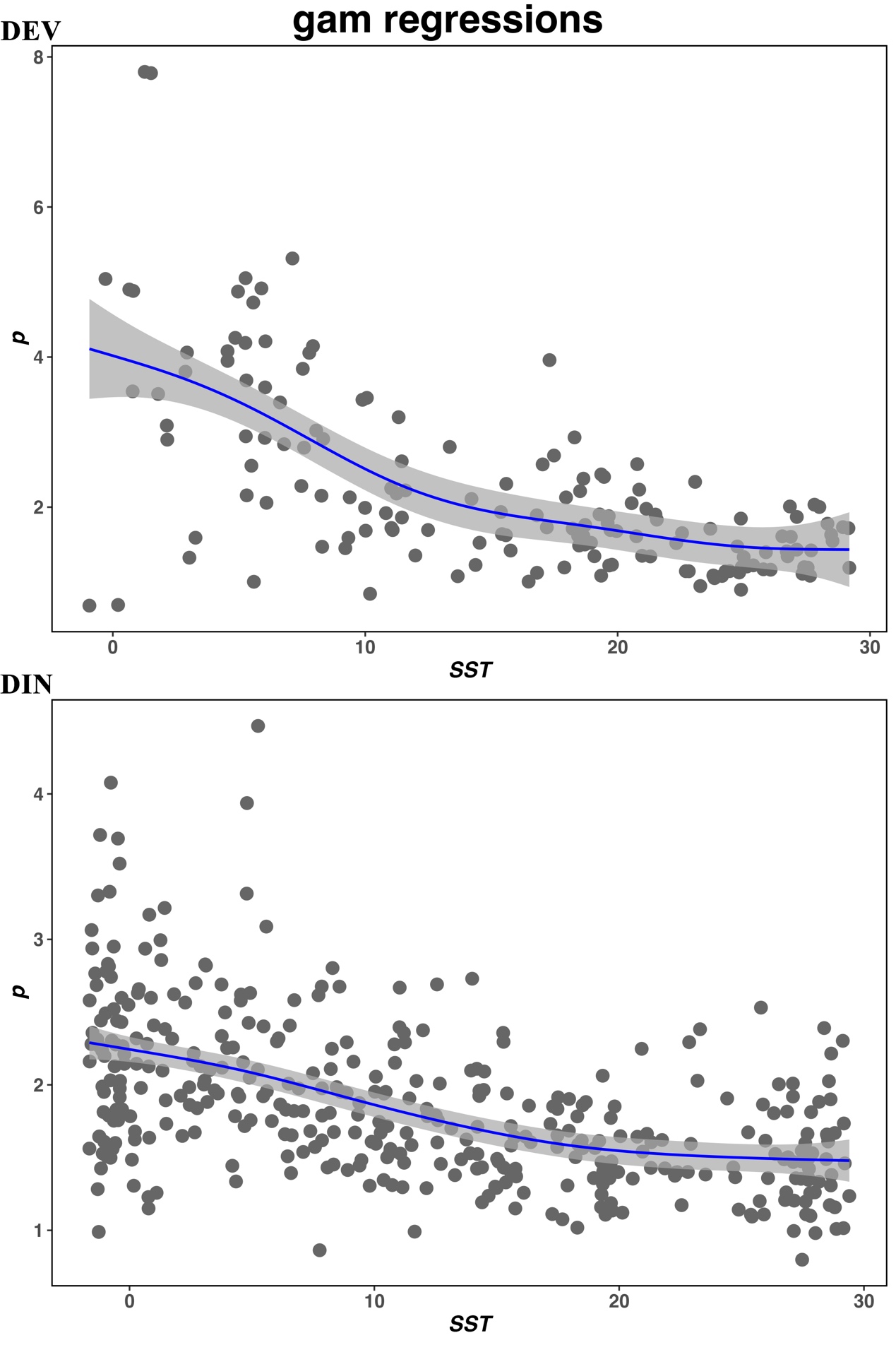

Supplement: Supplementary file 1 — Appendix S1 [file ECE3-12-e9584-s001.docx]
